# Diagnostic value of miR-186-5p for carotid artery stenosis and its predictive significance for future cerebral ischemic event

**DOI:** 10.1186/s13000-020-01007-w

**Published:** 2020-07-30

**Authors:** Weibo Lv, Tao Zhang, Hongwei Zhao, Shuang He, Bingwei Li, Yang Gao, Wenying Pan

**Affiliations:** 1 Department of Neurosurgery, Binzhou Medical University Hospital, Binzhou, 256603 Shandong China; 2 Department of Neurology, Affiliated Hospital of Nantong University, Nantong, 226001 Jiangsu China; 3 Department of Obstetrics & Gynecology, Binzhou Medical University Hospital, No. 661, Huanghe 2nd Road, Binzhou, 256603 Shandong China

**Keywords:** MiR-186-5p, Diagnosis, Asymptomatic CAS, Cerebral ischemic events

## Abstract

**Background:**

MicroRNAs (miRNAs) serve as novel promising biomarkers for the diagnosis and prognosis of many human diseases. This study investigated the diagnostic value of miR-186-5p for asymptomatic carotid artery stenosis (CAS), and its predictive value for future cerebral ischemic events (CIEs).

**Methods:**

Sixty-seven cases with asymptomatic CAS and 60 healthy individuals were recruited. Serum levels of miR-186-5p were tested by using qRT-PCR. Receiving–operator characteristic (ROC) curve was drawn based on sensitivity and specificity analyses. All asymptomatic CAS cases were followed up for 5 years. Kaplan-Meier method was applied for the evaluation of the predictive value of miR-186-5p for the occurrence of CIE.

**Results:**

The serum level of miR-186-5p was increased significantly in asymptomatic CAS patients. MiR-186-5p was the most significant factor associated with the high degree of carotid stenosis in asymptomatic CAS patients. In the ROC curve analysis, the AUC was 0.919, with the sensitivity of 89.6% and specificity of 81.7% at the cutoff value of 1.221. Kaplan-Meier method results revealed that high miR-186-5p level was associated with the occurrence of CIEs. High miR-186-5p level and high degree of carotid stenosis were independent factors for the occurrence of CIEs.

**Conclusion:**

MiR-186-5p serves as a potential diagnostic biomarker for patients with asymptomatic CAS, and predicts the occurrence of future CIEs.

## Introduction

Carotid artery stenosis (CAS) is a condition refer to the narrowing of one or both of the carotid arteries. It is usually induced by atherosclerotic lesions, and is considered to be one of the major causes of stroke and cerebral ischemic events (CIE) [1]. Research has shown that symptomatic CAS will increase the risk of stroke, furthermore, even if the CAS is asymptomatic, it still can place an individual at more than 3% increased risk of suffering from stroke in the next year [2, 3]. For the treatment of patients with CAS, the most important aspect is how to prevent an ischemic stroke and minimize related deaths. Therefore, it is important for the early diagnosis and timely intervention in patients with CAS, even though they are asymptomatic.

Many studies suggest that a variety of cellular processes such as proliferation, differentiation, and apoptosis can be regulated by microRNAs (miRNAs) [4, 5]. MiRNAs are a class of short non-coding RNA molecules, and play the functional role through targeting the target gene [6]. In recent years, close correlation between miRNAs and human diseases has been widely reported [7, 8]. There are many studies reporting the relationship between miRNAs and atherosclerotic diseases, and many miRNAs have been shown to play an important role in the development of CAS or CAS related diseases [9, 10]. MiR-186-5p is a highly conserved miRNA in mammals, and involved in the pathogenesis of various human diseases [11, 12]. Several studies suggest that miR-186-5p plays an important role in atherosclerotic disease, and as Wang et al. described, circulating miR-186-5p is a promising biomarker for the early diagnosis of acute myocardial infarction (AMI) [13, 14]. However, the role of miR-186-5p in the process of CAS or CIE remains unclear. It is interesting to investigate the clinical significance of miR-186-5p in CAS patients, which will help us to gain a better understanding of its value for the disease diagnosis and prognosis.

In the present study, we analyzed the expression changes of miR-186-5p in the serum of asymptomatic CAS patients by using quantitative reverse-transcription PCR (qRT-PCR). Considering the remarkable changes of serum miR-186-5p in asymptomatic CAS patients, we further investigated its clinical values for the diagnosis and prognosis of asymptomatic CAS.

## Materials and methods

### Study subjects and blood sampling

From January 2013 to May 2014, 67 cases diagnosed with asymptomatic CAS were enrolled, and another 60 healthy individuals were recruited as the control group. All study subjects gave written informed consents following the Declaration of Helsinki. The study protocols were approved by the Ethics Committee of Binzhou Medical University Hospital. All cases performed carotid artery color Doppler ultrasonography (CDUS), and the degree of CAS was determined according to the measurement results of computed tomography angiography (CTA). Patients who had conditions that can lead to stroke or CIE were excluded, including atrial fibrillation, myocardial infarction, cardiomyopathy, severe pulmonary disease, and arrhythmia. CAS refers to a > 50% stenosis of the extracranial internal carotid artery (ICA), and the stenosis severity was estimated according to the North American Symptomatic Carotid Endarterectomy Trial method [15]. An asymptomatic status was confirmed by a review of the patient’s history, a physical examination, and the numeric National Institutes of Health Stroke Scale [16]. The asymptomatic CAS patients had no history of stroke or transient ischemic attack, carotid revascularization, prior myocardial infarction, valvular heart disease, cardiomyopathy, severe pulmonary disease, arrhythmia. Among 67 cases with asymptomatic CAS, 36 cases had hypertension, 35 cases had diabetes and 38 cases had hypercholesterolemia. Hypertension was defined as the previous use of antihypertensive medications, a systolic pressure more than 140 mmHg or a diastolic pressure more than 90 mmHg for at least two separate measurements. Hypercholesterolemia was defined as total cholesterol at least 200 mg/dl. Diabetes was defined as a fasting blood glucose level of 126 mg/dL and above or current usage of oral antidiabetic drugs or insulin. Each control underwent carotid ultrasound to exclude critical CAS. Only individuals with normal Doppler ultrasound or evidence of carotid stenosis < 20% were included in the control group. All healthy controls had no history of diabetes, hypertension, hyperlipidemia, inflammatory diseases, cardiovascular diseases (CVDs), autoimmune disease and cancer.

Fasting blood samples were drawn from each individual, and serum samples were collected and stored at − 80 °C after centrifugation. The demographic and clinical information were recorded. Additionally,, all CAS patients were followed-up for 5 years to recorded the occurrence of CIEs, including strokes, transient ischemic attack (TIA) or sudden death. After the CIE occurs, the follow-up was ended.

### qRT-PCR assay

Firstly, total RNA was extracted from the serum of each subject by using TRIzol reagent (Invitrogen, Carlsbad, CA, USA) according to the manufacturer’s instructions. Briefly, the serum samples were homogenized in Trizol regnant, then chloroform was added to the 1/5 volume of Trizol and mixed thoroughly. The mixture was centrifuged at 12,000 g (4 °C) for 15 min. The supernatant was collected and mixed with isopropanol at a ratio of 1:1 and incubation for 10 min and was then centrifuged at 14,000 g (4 °C) for 10 min. The supernatant was removed and 100 μl 75% ethanol was added to the tubes and mixed thoroughly. After centrifugation at 8000 g (4 °C) for 5 min, the supernatant was removed and the tubes were air-dried. Then total RNA was reverse transcribed into complementary DNA by using a PrimeScript RT Reagent Kit (Takara, Tokyo, Japan). Then qRT-PCR was performed on a 7300 Real-Time PCR System (Applied Biosystems, USA) by using a SYBR green I Master Mix Kit (Invitrogen, Carlsbad, CA, USA). And relative levels of serum miR-186-5p were normalized by U6 by using 2^−ΔΔCt^ method.

### Statistical analysis

All statistical analyses were performed by using SPSS 21.0 software (SPSS Inc., Chicago, IL) and GraphPad Prism 7.0 software (GraphPad Software, Inc., USA). Categorical variables were presented as counts, and their comparisons between groups were analyzed by using Chi-Square test. Continuous variables were compared between groups by using Student’s t test. Receiving-operator characteristic (ROC) curve was drawn based on sensitivity and specificity analyses. Kaplan-Meier method was applied for the evaluation of the predictive value of miR-186-5p for the occurrence of CIE. The association of different variables with the degree of carotid stenosis was evaluated by using logistic regression analysis. Additionally, the Cox regression analysis was performed to assess independent predictive factors for CIE. The hazard ratios (HRs) or odd ratio (OR) and 95% confidence intervals (CIs) were calculated. Statistical significance was defined as *P* < 0.05.

## Results

### Clinical characteristics for CAS patients and healthy controls

As shown in Table 1, the clinical characteristics of the study subjects were recorded. It was shown that 60 healthy controls and 67 asymptomatic CAS patients were collected, with the mean age of 62.62 ± 9.66 and 61.57 ± 10.14 years old, respectively. No significant difference was detected for age, gender, BMI (body mass index), total cholesterol, HDL (high density lipoprotein), LDL (low density lipoprotein), triglycerides, fasting glucose, and SBP (systolic blood pressure) between the CAS patients and control groups (*P* > 0.05). But DBP (diastolic blood pressure) level was significantly higher in CAS patients group compared with the control group (*P* < 0.05).

**Table 1:** Comparison of the baseline data between healthy and asymptomatic CAS group

Characteristics	Healthy (*n* = 60)	Asymptomatic CAS (*n* = 67)	*P* values
Age (years)	62.62 ± 9.66	61.57 ± 10.14	0.553
Gender (male/female)	36/24	39/28	0.838
BMI (kg/m^2^)	25.11 ± 4.78	25.21 ± 4.22	0.907
Total cholesterol (mg/dL)	188.60 ± 13.15	193.57 ± 21.77	0.118
HDL (mg/dL)	44.92 ± 7.95	43.15 ± 8.27	0.223
LDL (mg/dL)	118.32 ± 12.31	122.46 ± 14.40	0.111
Triglycerides (mg/dL)	132.92 ± 18.07	138.28 ± 19.61	0.113
Fasting glucose (mg/dL)	109.15 ± 9.27	112.53 ± 12.92	0.080
SBP (mmHg)	122.13 ± 12.34	125.13 ± 21.83	0.336
DBP (mmHg)	80.37 ± 5.50	84.70 ± 10.78	0.005*

*CAS* Carotid artery stenosis, *BMI* Body mass index, *HDL* High density lipoprotein, *LDL* Low density lipoprotein, *SBP* Systolic blood pressure mmHg, *DBP* Diastolic blood pressure

*, *P* < 0.05

### Serum level of miR-186-5p is increased in CAS patients

**Fig. 1:**
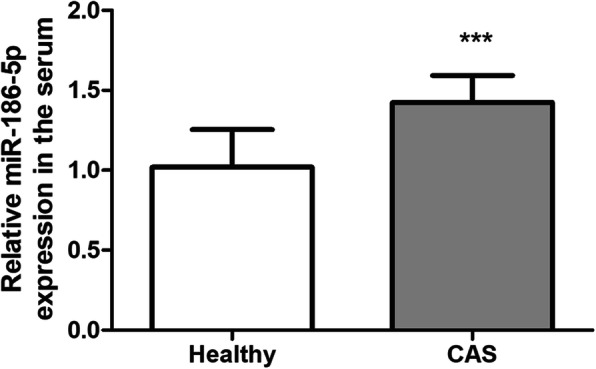
Serum level of miR-186-5p was increased significantly in asymptomatic CAS patients. *** *P* < 0.001

According to the results of qRT-PCR, the serum expression levels of miR-186-5p were compared between healthy individuals and asymptomatic CAS patients. It was found that the serum level of miR-186-5p was increased significantly in asymptomatic CAS patients (Fig. 1, *P* < 0.001).

### Comparison of the demographic, clinical, and biochemical properties between the groups

According to the mean value (1.423) of serum levels of miR-186-5p, all asymptomatic CAS patients were divided into low expression group (*n* = 31) and high expression group (*n* = 36). And the demographic, clinical, and biochemical properties were compared between the two groups. It was found that patients in the high miR-186-5p expression group had high levels of total cholesterol and a high degree of carotid stenosis compared with patients in low miR-186-5p expression group, and a larger proportion of cases suffered from hypertension were found in the high miR-186-5p expression group (Table 2). But for age, gender, BMI, HDL, LDL, triglycerides and fasting glucose, there was no significant difference between the two groups. These results revealed that miR-186-5p might be associated with dyslipidemia, hypertension, and degree of carotid stenosis in CAS patients.

**Table 2:** Association of miR-186-5p with the clinical data of asymptomatic CAS patients

Parameters	Low miR-186-5p expression group (< 1.423, *n* = 31)	High miR-186-5p expression group (≥1.423, *n* = 36)	*P* values
Age (years)	62.00 ± 10.25	61.19 ± 10.18	0.748
Gender (male/female)	19/12	20/16	0.635
BMI (kg/m^2^)	25.40 ± 4.35	25.03 ± 4.15	0.720
Total cholesterol (mg/dL)	186.65 ± 24.53	199.53 ± 17.30	0.015*
HDL (mg/dL)	42.19 ± 7.52	43.97 ± 8.89	0.384
LDL (mg/dL)	118.97 ± 15.15	125.47 ± 15.18	0.085
Triglycerides (mg/dL)	135.77 ± 20.33	140.44 ± 19.13	0.337
Fasting glucose (mg/dL)	110.26 ± 11.36	114.69 ± 13.95	0.163
Hypertension (Absence/Presence)	19/12	12/24	0.022*
Degree of carotid stenosis	61.90 ± 9.36	72.56 ± 9.77	0.000*

*CAS* Carotid artery stenosis, *BMI* Body mass index, *HDL* High density lipoprotein, *LDL* Low density lipoprotein

*, *P* < 0.05

### Association of different variables with the degree of carotid stenosis in CAS patients

The association of different variables with the degree of carotid stenosis was evaluated by using logistic regression analysis. It was observed that miR-186-5p (OR = 4.250, 95%CI = 1.200–15.057, *P* = 0.025) and total cholesterol levels (OR = 3.570, 95%CI = 1.083–11.766, *P* = 0.036) were the most significant factors associated with the high degree of carotid stenosis in CAS patients (Table 3).

**Table 3:** Association of different variables with the degree carotid stenosis in patients with asymptomatic CAS

Characteristics	OR	95%CI	*P* value
MiR-186-5p	4.250	1.200–15.057	0.025*
Age (years)	1.171	0.336–4.078	0.804
Gender (male/female)	1.750	0.483–6.346	0.394
BMI (kg/m^2^)	1.185	0.358–3.925	0.782
Total cholesterol (mg/dL)	3.570	1.083–11.766	0.036*
HDL (mg/dL)	1.172	0.249–5.507	0.841
LDL (mg/dL)	1.892	0.593–6.036	0.281
Triglycerides (mg/dL)	3.351	0.723–15.530	0.122
Fasting glucose (mg/dL)	1.388	0.419–4.602	0.592
Hypertension	2.034	0.600–6.893	0.254

*CAS* Carotid artery stenosis, *BMI* Body mass index, *HDL* High density lipoprotein, *LDL* Low density lipoprotein, *SBP* Systolic blood pressure mmHg, *DBP* Diastolic blood pressure, *OR* Odd ratio, *95%CI* 95% confidence interval

*, *P* < 0.05

### Diagnostic value of miR-186-5p for asymptomatic CAS

**Fig. 2:**
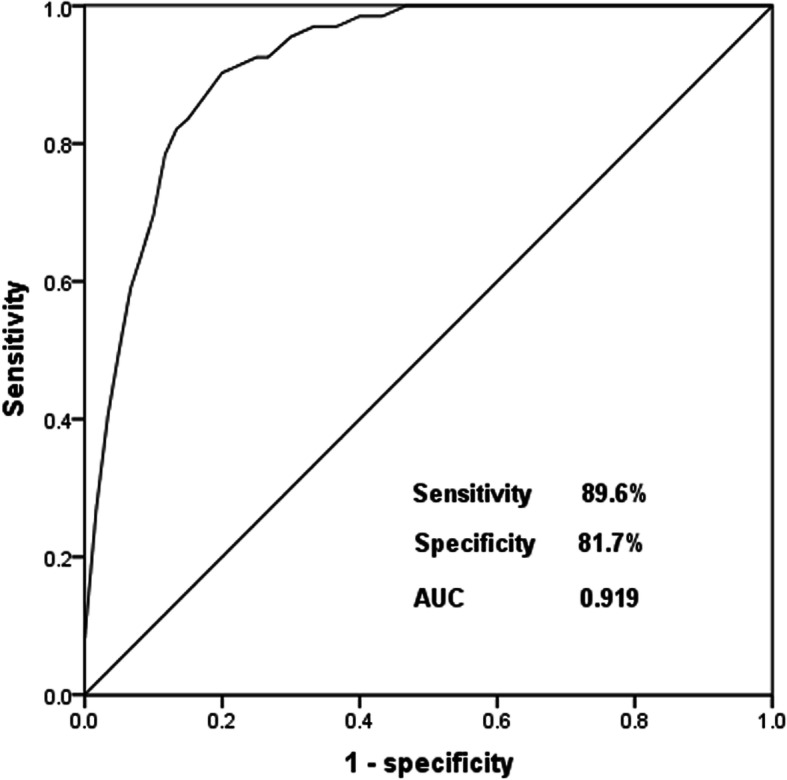
ROC curve was constructed to evaluate the diagnostic value of miR-186-5p for asymptomatic CAS. In the ROC curve analysis, the AUC was 0.919, with the sensitivity of 89.6% and specificity of 81.7% at the cutoff value of 1.221

According to the serum levels of miR-186-5p in both asymptomatic CAS patients and healthy controls, the ROC curve was constructed to evaluate the diagnostic value of miR-186-5p for asymptomatic CAS. In the ROC curve analysis, the AUC (area under the curve) was 0.919, with the sensitivity of 89.6% and specificity of 81.7% at the cutoff value of 1.221 (Fig. 2). The data indicated that serum miR-186-5p could distinguish asymptomatic CAS patients from healthy controls.

### Predictive value of miR-186-5p for the occurrence of CIE in asymptomatic CAS patients

**Fig. 3:**
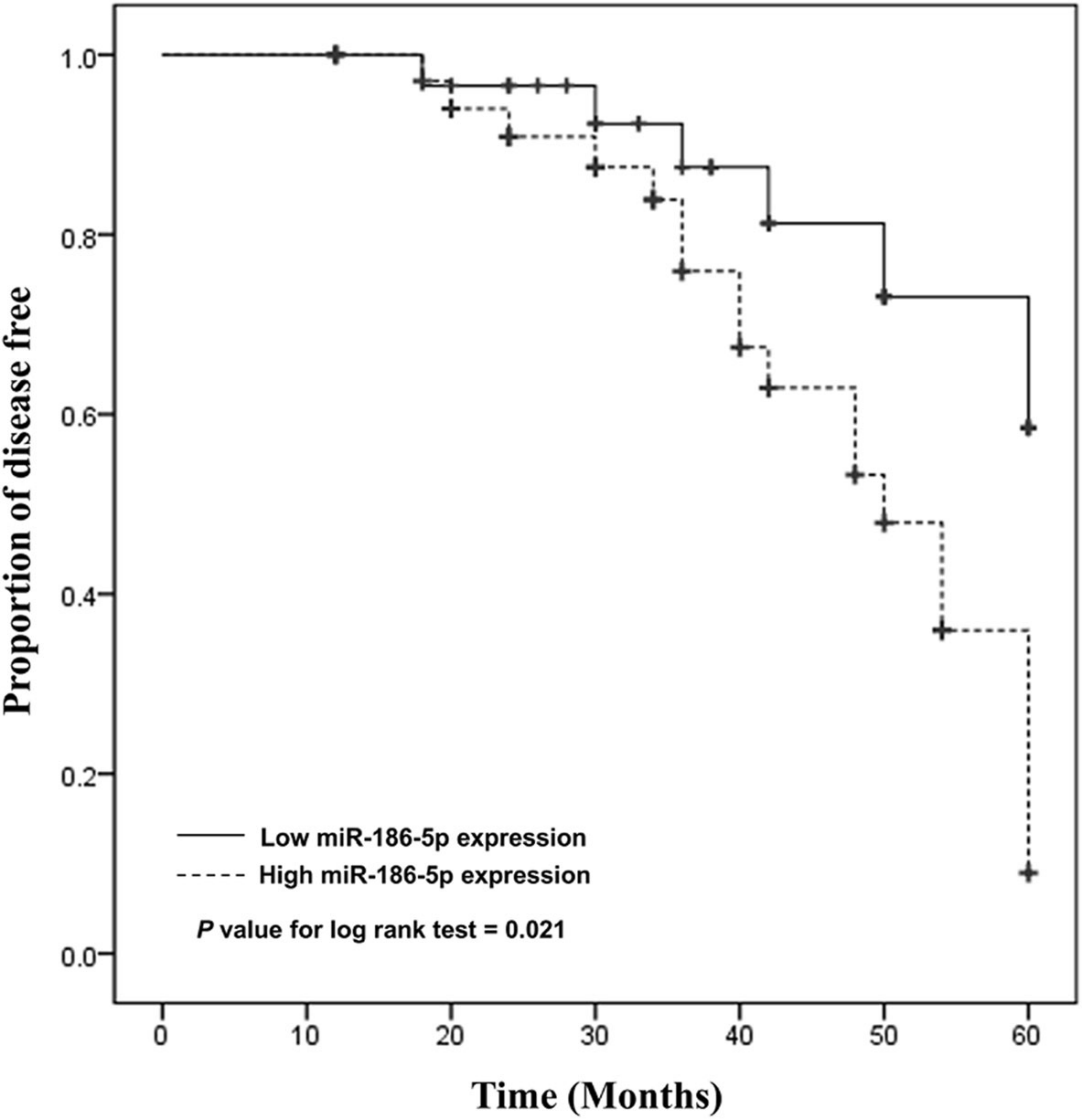
Kaplan-Meier method was applied for the evaluation of the predictive value of miR-186-5p for the occurrence of CIE in asymptomatic CAS patients. It was observed that high miR-186-5p level was associated with the occurrence of CIEs

According to the 5-year follow-up data, the Kaplan-Meier method was applied for the evaluation of the predictive value of miR-186-5p for the occurrence of CIE in asymptomatic CAS patients. All patients completed the fiver year follow-up, and among 67 asymptomatic CAS patients, 24 cases underwent CIE, including 19 TIAs and 5 strokes. Among 24 CIE patients, 18 cases were at high miR-186-5p expression and 6 were at low miR-186-5p expression. Kaplan-Meier method results revealed that high miR-186-5p level was associated with the occurrence of CIEs (Fig. 3). Multivariate Cox regression analysis was performed for all the variables measured in the study, including age, gender, BMI, dyslipidemia, diabetes, hypertension, degree of carotid stenosis and serum miR-186-5p expression. The results suggested that high miR-186-5p level (HR: 4.190, 95% CI: 1.166–15.061, *P* = 0.028) and high degree of carotid stenosis (HR: 3.143, 95% CI: 1.117–8.845, *P* = 0.030) were independent factors for the occurrence of CIEs in asymptomatic CAS patients (Table 4).

**Table 4:** Multivariate Cox regression analysis for miR-186b-5p in patients with asymptomatic CAS

Characteristics	HR	95%CI	*P* value
MiR-186-5p	4.190	1.166–15.061	0.028*
Age (years)	1.486	0.550–4.014	0.434
Gender (male/female)	1.041	0.412–2.630	0.932
BMI (mg/dL)	1.946	0.723–5.236	0.187
Total cholesterol (mg/dL)	1.802	0.590–5.501	0.301
HDL (mg/dL)	1.840	0.524–6.464	0.341
LDL (mg/dL)	1.349	0.552–3.298	0.512
Triglycerides (mg/dL)	3.059	0.677–13.817	0.146
Fasting glucose (mg/dL)	1.208	0.488–2.992	0.683
Hypertension	1.959	0.745–5.149	0.173
Degree of carotid stenosis	3.143	1.117–8.845	0.030*

*CAS* Carotid artery stenosis, *BMI* Body mass index, *HDL* High density lipoprotein, *LDL* Low density lipoprotein, *HR* Hazard ratio, 95%CI, 95% confidence interval

*, *P* < 0.05

## Discussion

The present study determined that miR-186-5p was overexpressed in the serum of patients with asymptomatic CAS. Serum miR-186-5p might be a promising biomarker for the early diagnosis of asymptomatic CAS, it could distinguish asymptomatic CAS patients from healthy controls. Additionally, overexpression of miR-186-5 was associated with the high degree of carotid stenosis and the occurrence of CIE, it might be a promising biomarker for predicting the risk of future CIE in asymptomatic CAS patients.

CAS is a slow but gradual process, and the incidence of CAS is gradually increasing. However, for patients with asymptomatic CAS, there is always no obvious symptom clinically, which may influence the early diagnosis. Traditionally, CAS is usually detected by using some imaging procedures, such as MR angiography (MRA) and conventional transcranial Doppler (TCD) sonography [17]. Recently, miRNAs have been proved to exist stably in human plasma, and play an important role in the regulation of a variety of developmental and pathological processes [18, 19]. Previous evidence has suggested that miRNAs serve as novel promising biomarkers for the diagnosis and prognosis of many human diseases, including CAS [9]. Recently, Dolz et al. tried to construct specific circulating miRNA expression profiles in asymptomatic CAS patients by using Affymetrix microarrays, and several miRNAs were determined to be aberrantly expressed by using qRT-PCR, including miR-199b-3p, miR-27b-3p, miR-130a-3p, miR-221-3p, and miR-24-3p [9]. Another study by Badacz et al. reported that several miRNAs were differentially expressed in symptomatic CAS patients compared with asymptomatic cases, the dysregulation of miRNAs showed a close association with plaque morphology and structure and might be potential prognostic factors for future cardiovascular events [20].

In the present study, we compared the expression level of miR-186-5p in the serum of 67 asymptomatic CAS patients with 60 healthy controls. Analysis of the expression levels of miR-186-5p between two groups showed that miR-186-5p was significantly overexpressed in asymptomatic CAS patients compared with healthy controls, indicating that miR-186-5p might play a crucial role in the occurrence and development of CAS. Consistently, as previous research reported, plasma miR-186-5p shows a remarkable increase in acute coronary syndromes (ACS) patients compared with that in cardiovascular disease (CAD) patients, suggesting the potential role of miR-186-5p in the comprehensive risk stratification of atherosclerotic cardiovascular diseases [21]. These data revealed the important role of miR-186-5p in the progression of atherosclerosis-related cardiovascular and cerebrovascular diseases. Additionally, the logistic regression analysis results revealed that the miR-186-5p level was the most significant factor associated with the high degree of carotid stenosis in CAS patients. It was concluded that miR-186-5p might be associated with the severity of CAS.

MiR-186-5p plays a crucial role in a variety of human diseases, and many studies focus on its clinical value for disease diagnosis and prognosis [13, 22]. MiR-186-5p has been assessed as a possible biomarker for the early diagnosis and prediction of several types of cancers, such as nonmelanoma skin cancer (NMSC) and head and neck squamous cell cancer (HNSCC) [23, 24]. Additionally, in AMI patients, circulating miR-186-5p was reported to be highly expressed in the early stage of AMI and closely associated with the level of cardiac troponin I (cTnI), the results indicated that miR-186-5p showed considerable diagnostic efficiency for predicting AMI [13]. Given the significant changes of serum miR-186-5p in patients with asymptomatic CAS, the ROC curve was constructed to evaluate the diagnostic value of miR-186-5p for asymptomatic CAS. The results indicated that serum miR-186-5p could distinguish asymptomatic CAS patients from healthy controls, it might be a promising biomarker for CAS diagnosis. But our study sample is relatively small, other studies with a larger population are needed to verify the present results. Besides, according to the 5-year follow-up data, we also evaluate the predictive value of miR-186-5p for the occurrence of CIE in asymptomatic CAS patients. The Kaplan-Meier and Multivariate Cox regression analysis results demonstrated that high miR-186-5p level was an independent factor for the occurrence of CIEs in asymptomatic CAS patients. However, during the follow-up time, the lifestyle and medication condition information were not included systematically, which might result in the results bias, thus more studies are needed to verify the present results.

Clinically, analysis of the demographic and clinical data results demonstrated that a larger proportion of cases with high total cholesterol levels or suffer from hypertension were found in the high miR-186-5p expression group, suggesting that higher miR-186-5p level might be associated with dyslipidemia and hypertension in asymptomatic CAS patients. Hypertension is a powerful risk factor for cardiovascular and cerebrovascular diseases, which are important causes of mortality and disability in the elderly [25]. A recent study has found that the plasma level of miR-186-5p was higher in hypertensive patients than in healthy subjects, and miR-186-5p may be related to the occurrence of hypertension [26]. Consistently, the current data indicated that higher miR-186-5p levels might be associated with the occurrence of hypertension in asymptomatic CAS patients. Dyslipidemia has been recognized as an important risk factor for the pathogenesis and development of CAS [27]. In the present study, the results of the clinical data analysis suggested that miR-186-5p might be associated with dyslipidemia in asymptomatic CAS patients. Additionally, miR-186-5p has been reported to play an important role in atherosclerotic diseases, and it is also determined to promote macrophage lipid accumulation [14]. From these results, we concluded that the regulation effect on dyslipidemia and hypertension might be the possible underlying mechanism for the crucial role of miR-186-5p in CAS. However, further studies are needed to determine the possible mechanism of miR-186-5p in the pathogenesis of CAS.

## Conclusion

In summary, the present study highlighted the potential role of serum miR-186-5p for the early diagnosis of asymptomatic CAS. Additionally, overexpression of miR-186-5p was closely associated with the occurrence of CIEs for asymptomatic CAS patients. Collectively, the present results indicated that miR-186-5p serves as a potential diagnostic biomarker for patients with asymptomatic CAS and predicts the occurrence of future CIEs.

## Data Availability

The datasets used and/or analysed during the current study are available from the corresponding author on reasonable request.

## Abbreviations

miRNAs: microRNAs
CAS: Carotid artery stenosis
CIEs: Cerebral ischemic events
ROC: Receiving–operator characteristic
AMI: Acute myocardial infarction
qRT-PCR: Quantitative reverse-transcription PCR
CDUS: Color Doppler ultrasonography
CTA: Computed tomography angiography
TIA: Transient ischemic attack
HRs: Hazard ratios
CIs: Confidence intervals
MRA: MR angiography
TCD: Transcranial Doppler
ACS: Acute coronary syndromes
CAD: Cardiovascular disease
NMSC: Nonmelanoma skin cancer
HNSCC: Head and neck squamous cell cancer
cTnI: Cardiac troponin I
